# The British Medical Association

**Published:** 1892-08-06

**Authors:** 


The Hospital
An Institutional Journal of
Science, Medicine, Nursing, and Philanthropy.
*For Hospitals, Asylums, Infirmaries, Dispensaries, Medical Practitioners, Students, Nurses, and the Charitable Public.*
Vol. XII.—No. 306.] [Aug. 6, 1892.

### THE BRITISH MEDICAL ASSOCIATION.

In the summer of 1832 the late Sir Charles Hastings summoned a meeting of the medical men in practise in Worcester and the neighbouring counties to the Board Room of the Worcester Infirmary, to inaugurate the Provincial Medical and Surgical Association. By 1857, when the association assembled at Nottingham, it possessed two thousand members, and there was an attendance of about ninety at this meeting. Last week, under a new name—that of the British Medical Association—the medical congress met once more at Nottingham, and the roll of members included the names of upwards of fourteen thousand medical practitioners, of whom a very large number were present. The chief factor in this triumph of organization has been the success of the *British Medical Journal*, under the editorship of that singularly able and brilliant journalist, Mr. Ernest Hart, to whose organizing and administrative gifts, combined with indomitable pluck and perseverance, the British Medical Association owes more than to, probably, anything else. The *Journal* holds a deservedly high and, indeed, a foremost place in medical journalism, not only in the British Empire but throughout the world. It is generally first with all medical news, and its scientific work is most ably done. It is officially stated—and we can quite believe it—that during the past year by far the larger part of the 984 new members have, as usual, joined as an immediate consequence of the half-yearly issue of a specimen number of the *Journal* to members of the profession outside the ranks of the association, which is thus shown to be the chief recruiting agent for membership. Apart from its medical, scientific, and financial value, the *British Medical Journal* and its editor have done much to promote fruitful legislation and the progressive advance of sound opinion, lay and medical, on many subjects allied to the public health and the general welfare of doctors, nurses, and the people at large. The impartial and disinterested policy pursued by the conductors of the *British Medical Journal* makes it deservedly the most trusted of all professional newspapers.

For thirty years Mr. Ernest Hart has been foremost in arousing and fixing the attention of statesmen and politicians whenever Parliamentary influence was necessary to secure the well-being of members of the profession or the public. He it was who laboured so successfully to secure the introduction and passing of Hardy's Act, whereby the poor law infirmaries were established in London upon their present satisfactory basis. Medical men in the army and navy owe much to his self denying and persistent labours, and during the past year the Irish Poor Law Service has been similarly aided by Mr. Hart's wise counsel and influence. It would be impossible in the space at our command to do justice to the vast public work of Mr. Ernest Hart, but it is not too much to say that any Prime Minister who advised the Queen to confer a substantial honour upon one to whom his day and generation owe so much would not only win thereby wide popularity, but would also do substantial justice to a man of the day whose great public services have been allowed to remain too long without high official recognition and reward. Our colleagues of the lay press know full well the peculiar value and importance of the public services rendered by Mr. Ernest Hart, and we have full confidence that they will cordially endorse the views we have expressed on the subject.

It is interesting to recall the original objects which the early founders of the British Medical Association set themselves to accomplish sixty years ago. They were, as the president, Mr. Joseph White, pointed out, briefly as follows :—

The collection of important medical and surgical knowledge arising from public and private practice in the great provincial centres and in rural districts ; increase of knowledge of the medical topography of England, in its widest sense, and which could best be collected by those resident on the spot; investigations of the modifications of endemic and epidemic diseases in different situations and at various periods, so as to trace their connection with peculiarities of soil and climate, or with the localities, habits, and occupations of the people; and, perhaps most important of all, the maintenance of the honour and dignity of the profession generally by promoting friendly intercourse and free communication amongst its members, and by establishing between them that harmony and good feeling which ought ever to characterise a liberal profession.

These important matters have been kept steadily in view from the commencement, and the success which has attended the efforts to carry them out successfully has made the British Medical Association one of the most powerful organizations of professional men the world has ever produced. The branches of the association now extend over nearly every part of the world, its members collectively labour in almost every climate, and so include every variety of men for the study and observation of every variation which these climates originate or produce. More important still, the fifteen thousand medical observers which the members represent "are all engaged in the same pursuits, are all animated by the same aim and desires for the same kind of knowledge, and all have an opportunity of meeting in annual congress for the communication and discussion" of the results which may be achieved. Surely such an association can justly claim that its members possess "an unequalled opportunity for developing in the highest possible way the fruits of this combined work" on which all will agree with Mr. White the future of medical science must in a large measure depend. There is no congress out of the many now held in various countries during each year which can exceed the importance that attaches to the annual meetings of the British Medical Association. It has become an honour to be enrolled upon its list of members, and we rejoice that this year's meeting unanimously resolved to admit legally qualified women as members of so august and important a body.

The Association has, however, social as well as scientific ends in view. It fulfils a most useful purpose by enabling old friends to meet and new friendships to be formed. Knowledge is thus given and imparted; men of note are enabled to impress the weight of their conviction and the fruits of their labours upon their brother scientists by that most potent of all influences—personal intercourse and intercommunication. For the greater portion of each twelve months this army of busy professional men has to labour apart, to pursue anxious inquiries, and to engage in deep research either individually or, at the best, in small groups. It is not difficult nowadays to convey the results so achieved to the world at large by means of the printing press and the public journals, but after these results have been studied and examined, it is of immense advantage to science and to the individual worker, that they should be submitted to the further test of public discussion by experts who have devoted the best part of their lives to the mastery of the principles upon which all true science must be based. In this way a real community of interest is established, and by these annual meetings of medical men the truest sympathy is promoted between the man of the laboratory and the practical physician, who is dependent for most of the results he may providentially be permitted to secure in his fight against disease and death, on the ever-increasing knowledge placed at his disposal by the zealous student of research. The public owe much to medicine and her votaries, and for this reason the meetings of the British Medical Association excite an increasing interest amongst all classes of the people, who are beginning to learn and to appreciate the importance of the proceedings, not only to science, but to their families and to themselves.

